# Neuroprotective Effects of Water Extract from Brown Algae *Petalonia binghamiae* in an Experimental Model of Focal Cerebral Ischemia In Vitro and In Vivo

**DOI:** 10.3390/cimb45100531

**Published:** 2023-10-17

**Authors:** Sun Ho Eom, Geum-Lan Hong, Hyun Bae Kang, Nam-Seob Lee, Do Kyung Kim, Young Gil Jeong, Chun-Sung Kim, Yung Choon Yoo, Bong Ho Lee, Ju-Young Jung, Dong-Sub Kim, Seung Yun Han

**Affiliations:** 1 Healinnols Inc., Daejeon 34054, Republic of Korea; always703@naver.com (S.H.E.); khb0425@healinnols.com (H.B.K.); 2 Department of Anatomy, College of Medicine, Konyang University, Daejeon 35365, Republic of Koreanslee@konyang.ac.kr (N.-S.L.); dokyung@konyang.ac.kr (D.K.K.); ygjeong@konyang.ac.kr (Y.G.J.); 3 Department of Oral Biochemistry, College of Dentistry, Chosun University, Gwangju 61452, Republic of Korea; cskim2@chosun.ac.kr; 4 Department of Microbiology, College of Medicine, Konyang University, Daejeon 35365, Republic of Korea; yc_yoo@konyang.ac.kr; 5 Department of Chemical Technology, Hanbat National University, Daejeon 34158, Republic of Korea; ibh011@hanbat.ac.kr; 6 Department of Histology & Institute of Veterinary Science, College of Veterinary Medicine, Chungnam National University, Daejeon 34134, Republic of Korea; 7 Division of Natural Product Research, Korea Prime Pharmacy Co., Ltd., Gwangju 61473, Republic of Korea; ds.kim@koreaprime.co.kr

**Keywords:** brown algae, *Petalonia binghamiae*, focal cerebral ischemia, middle cerebral artery occlusion and reperfusion, heme oxygenase-1

## Abstract

Focal cerebral ischemia (fCI) can result in brain injury and sensorimotor deficits. Brown algae are currently garnering scientific attention as potential therapeutic candidates for fCI. This study investigated the therapeutic effects of the hot water extract of *Petalonia binghamiae* (wPB), a brown alga, in in vitro and in vivo models of fCI. The neuroprotective efficacy of wPB was evaluated in an in vitro excitotoxicity model established using HT-22 cells challenged with glutamate. Afterward, C57/BL6 mice were administered wPB for 7 days (10 or 100 mg/kg, intragastric) and subjected to middle cerebral artery occlusion and reperfusion (MCAO/R) operation, which was used as an in vivo fCI model. wPB co-incubation significantly inhibited cell death, oxidative stress, and apoptosis, as well as stimulated the expression of heme oxygenase-1 (HO-1), an antioxidant enzyme, and the nuclear translocation of its upstream regulator, nuclear factor erythroid 2-related factor 2 (Nrf2) in HT-22 cells challenged with glutamate-induced excitotoxicity. Pretreatment with either dose of wPB significantly attenuated infarction volume, neuronal death, and sensorimotor deficits in an in vivo fCI model. Furthermore, the attenuation of oxidative stress and apoptosis in the ischemic lesion accompanied the wPB-associated protection. This study suggests that wPB can counteract fCI via an antioxidative effect, upregulating the Nrf2/HO-1 pathway.

## 1. Introduction

Cerebral ischemia, a leading cause of mortality or disability worldwide, is categorized into global and focal cerebral ischemia (fCI), with the latter being more prevalent among patients with cerebral ischemia [1,2]. fCI occurs when the cerebral artery supplying a specific area of the brain becomes obstructed, resulting in oxygen and nutrient deprivation, neuronal apoptosis, and eventual death [3,4,5,6]. Current therapeutics for fCI aim to restore cerebral blood flow within 3.5 h to prevent further brain tissue damage, but reperfusion can lead to reperfusion injury due to the activation of oxidizing enzymes that generate reactive oxygen species (ROS) [7,8].

Oxidative stress, resulting from the imbalance between ROS accumulation and antioxidant defense systems, has been extensively studied in the pathology of fCI [9,10]. It triggers blood–brain barrier leakage and infiltration of inflammatory cells into the brain parenchyma [11,12]. Additionally, ROS play a role in fCI pathogenesis by inducing mitochondria-dependent apoptosis through the release of cytochrome C from the mitochondria [13]. Thus, targeting oxidative stress could be a potential therapeutic and preventive strategy for patients with fCI.

In parallel with the above context, several synthetic drugs have been developed to scavenge ROS in order to address intracellular ROS levels in pharmaceutical intervention [14,15,16]. However, synthetic ROS scavengers encounter challenges such as poor stability, high toxicity, and low bioavailability [17]. Issues with aqueous solubility, ease of metabolism, lack of selectivity, and systemic adverse reactions have hindered their use [18].

Considering that stability, nontoxicity, and bioavailability are the ultimate standards for any medicine, there is currently growing scientific interest in potential drugs that can mitigate the drawbacks associated with synthetic counterparts by leveraging their antioxidative role. Marine species have evolved secondary metabolic pathways to produce molecules that enable them to adapt to environmental stressors, including oxidative stress, extreme temperatures, and photodynamic damage [19,20]. Consequently, marine organisms are able to protect themselves from oxidative damage despite environmental changes due to the presence of bioactive molecules [21]. Terpenes, peptides, alkaloids, and various uncharacterized structures extracted from marine organisms have demonstrated diverse health-promoting effects, such as antibacterial, antiviral, antidiabetic, anticoagulant, antihypertensive, anticancer, anti-inflammatory, and antioxidant properties [22,23,24,25].

*Petalonia binghamiae* (PB) is a type of brown alga that is primarily found in coastal areas of the western United States, Republic of Korea, and Japan, which has traditionally been consumed as an edible seaweed in fishing communities [26]. PB has multiple leaves that are approximately 25–30 mm wide and 250–300 mm long [27]. Previous studies have reported various beneficial properties of PB, including antidiabetic, anti-inflammatory, and antioxidant activities [28,29,30]. Notably, a recent study demonstrated that PB protected mouse-derived myoblast cells against oxidative stress by upregulating the expression of heme oxygenase-1 (HO-1), a major antioxidant enzyme, and nuclear factor erythroid 2-related factor 2 (Nrf2), an upstream transcriptional regulator [29].

Based on the fact that the imbalance between ROS accumulation and the protective systems mediated by antioxidant enzymes is a key factor in the pathogenesis of fCI, it is plausible that consumption of PB could confer protection against fCI. However, to the best of our knowledge, the effects of PB on fCI have not previously been investigated. Therefore, this study aimed to evaluate the neuroprotective effects of PB extract in an in vitro neuronal cell line (HT-22) challenged with glutamate-induced excitotoxicity, as well as an in vivo mouse model of fCI, using middle cerebral artery occlusion and reperfusion (MCAO/R) operation.

## 2. Materials and Methods

### 2.1. Preparation of PB Extracts

In May 2021, PB was harvested from Seoguipo-si on Jeju Island. To prepare an extract using solvent extraction, a four-gram portion of the dried PB sample was finely ground. It was then soaked separately in 100 mL of two different solvents: water and ethanol. This maceration process occurred in darkness over a 24 h period, with periodic shaking to enhance extraction efficiency. After incubation, the solution was carefully filtered using Whatman filter paper No. 4, which has a pore size ranging from 20 to 25 µm, to maintain hygiene standards. Subsequently, the remaining moist powder underwent a second round of extraction in the respective solvents. This second extraction involved sporadic shaking over a 12 h period, followed by filtration to maximize the sample’s yield. The resulting solutions were one derived from water extraction (wPB) and the other from 70% ethanolic extraction (ePB). These solutions were obtained after 1 h of shaking at a temperature of 24 °C. They were then subjected to centrifugation at 2250× *g* for 10 min and subsequently dried using a freeze dryer (VaCo2, Niedersachsen, Germany) at −83 °C. The resulting lyophilized powders from the wPB and ePB solutions exhibited dark brown and dark green colors, respectively.

### 2.2. Cell Culture and Treatment

The HT-22 cell line, a type of mouse hippocampal neuronal cell, was obtained from Millipore (Cat# SCC129, RRID: CVCL_0321; Billerica, MA, USA) for this study. HT-22 cells were cultured in Dulbecco’s modified Eagle’s medium (DMEM; Gibco, MA, USA) supplemented with 10% fetal bovine serum (Gibco) and 1% penicillin and streptomycin (Gibco) at 37 °C in a 5% CO_2_ environment, with medium replacement every 48 h. After confirming the higher biocompatibility of wPB compared to ePB, wPB was chosen for further in vitro experiments. Furthermore, after confirming an approximate median lethal dose (LD_50_) of 24 h of treatment with glutamate as 3 mM, this dosage was applied in our in vitro model. The cells were subjected to four different treatments and labeled as follows: no treatment, the control (CTRL) cells; 3 mM glutamate for 24 h, the GLU cells; and coincubation with 10 or 100 μg/mL wPB and 3 mM glutamate for 24 h, the GLU + wPB10 or GLU + wPB100 cells, respectively.

### 2.3. Liquid Chromatography–Mass Spectrometry (LC-MS)

After confirming the higher biocompatibility of wPB compared to ePB, we identified the chemical compositions of wPB in accordance with our previous protocol [31]. In summary, an LC–MS instrument (Time-of-Flight/6500 series, Agilent, Palo Alto, CA, USA) connected to a high-performance liquid chromatography (HPLC) system (1290 Infinity Binary Pump, Agilent) with an electrospray ionization (ESI) interface was utilized for chemical profiling. Chromatographic separations were carried out using a C18 column (5 µm, 150 mm × 2.1 mm; ZORBAX Eclipse Plus C18, Agilent) at a flow rate of 0.2 mL/min, with two mobile phases. The gradient system consisted of high A for the first 3 min, high B for the next 15 min, and then high A again (A: 0.1% formic acid in water and B: 0.1% formic acid in acetonitrile). The column temperature was maintained at 40 °C, and the injection volume was 3 µL, with a total run time of 30 min. Spectra were obtained in ESI- modes using a photodiode array detector. An Agilent Database Library was used for compound verification.

### 2.4. Cell Viability Assay

Cell viability was assessed by using the 3-(4,5-dimethyl-2-thiazolyl)-2,5-diphenyl-2H-tetrazolium bromide (MTT) reagent (Sigma-Aldrich, St. Louis, MO, USA) following the manufacturer’s instructions. HT-22 cells were seeded at a density of 1 × 10^4^ cells per well in a 96-well plate and incubated for 24 h. Subsequently, after various treatments (see Section 2.2 for details), the well plates were replaced with phosphate-buffered saline (PBS) containing 5 μg/mL of MTT and further incubated at 37 °C for 4 h. The absorbance was measured at 520 nm using an ELx808 microplate reader (BioTeck Instruments, Winooski, VT, USA). The percentage of cell viability was calculated by comparing the results with the CTRL group.

### 2.5. ROS Measurement

Intracellular ROS were quantified using the fluorogenic probe, 2′,7′–dichlorodihydrofluorescein diacetate (DCF-DA) assay (Sigma-Aldrich), according to the manufacturer’s protocol. Cells were seeded in a 12-well plate at a density of 1 × 10^5^ cells/well and incubated for 24 h. After different treatments (see Section 2.2 for details), the well plates were replaced with 100 mM DCF-DA diluted in DMEM for 30 min. The cells were washed with fresh DMEM and incubated for an additional 10 min to allow for excitation of DCF fluorescence. The resulting fluorescence was photographed and recorded with a fluorescence analyzer (JuLI-FL, Pleasanton, CA, USA) at an excitation/emission spectra of 504/524 nm. The intensities were analyzed using ImageJ software (ImageJ v1. 46a; NIH, Bethesda, MD, USA) and calculated as the fold change in the CTRL group.

### 2.6. Immunocytochemistry

The immunocytochemistry was conducted according to previously described protocols [32,33]. Cells were seeded on coverslips in 12-well plates at a density of 1 × 10^5^ cells/well and incubated for 24 h. Subsequently, after different treatments (see Section 2.2 for details), cells were washed with PBS, fixed with 100% ethanol, and treated with a blocking solution of 3% bovine serum albumin diluted in PBS for 30 min. Cells were incubated with rabbit anti-Nrf2 (Abcam, Cambridge, UK) diluted in the blocking solution (1:200) for 24 h, followed by washing and incubation with Cy2-conjugated anti-rabbit IgG (Invitrogen, Carlsbad, CA, USA) diluted in PBS (1:200). After further PBS washing, cells were treated with 4′, 6-diamidino-2-phenylindole (DAPI)-conjugated anti-fade mounting medium (Abcam) and visualized using laser scanning confocal microscopy (LSM-700; Carl Zeiss, Oberkochen, Germany). Cells displaying nuclear Nrf2 were quantified (>100 cells per cell group, 400×) and expressed as a percentage relative to the CTRL group.

### 2.7. Western Blot

The experiment involved seeding cells in a 6-well plate at a density of 1 × 10^6^ cells/well, followed by a 24 h incubation period. After various treatments (see Section 2.2 for details), the cells were harvested, and cell lysates were obtained using lysis buffer (Pro-Prep™, iTrRON Biotechnology, Seongnam, Republic of Korea). The total protein concentration in the supernatant was determined using bicinchoninic acid protein assay (Pierce Biotechnology, Rockford, IL, USA). Next, the protein sample (30 µg/mL) was separated using 10% SDS-PAGE and transferred onto a polyvinylidenedifluoride (PVDF) membrane (Bio-Rad, Hercules, CA, USA), which was blocked with 5% skim milk in Tris-buffered saline with 0.1% Tween 20 (TBS-T) for 2 h at 24 °C. The membranes were incubated with rabbit anti-HO-1 (1:1000, Abcam), and β-actin (1:2000, Sigma-Aldrich) diluted in TBS-T, for 24 h at 4 °C. Proteins were subsequently incubated with horseradish peroxidase (HRP)-conjugated anti-rabbit IgG (1:1000; Vector Laboratories, Burlingame, CA, USA) diluted in PBS for 1 h at 36 °C. For the subcellular quantification of Nrf2, a subcellular fractionation kit (Thermo-Fisher Scientific, Waltham, MA, USA) was used following the manufacturer’s protocol. The protocols for determining the total protein concentration, SDS-PAGE separation, and protein transfer to PVDF membranes were identical to those above-mentioned. The membranes were then incubated with the rabbit anti-Nrf2 (1:1000, Abcam) diluted in TBS-T for 24 h at 4 °C, followed by incubation with HRP-conjugated anti-rabbit IgG (Vector Laboratories) for 1 h at 36 °C. The quality of the nuclear and cytosolic fractionation was confirmed by immunoblotting using rabbit anti-Lamin B (1:1000, Sigma-Aldrich) and β-actin (1:2000, Sigma-Aldrich), respectively. After five washes with PBS, the proteins on the PVDF membranes were detected using a chemiluminescence detection system (Amersham ECL Prime Western Blotting Detection Reagent; GE Healthcare Life Sciences, Little Chalfont, UK), as per the manufacturer’s protocol. The resulting bands were photographed using a Davinch-Chemi imaging device (Davinch-K; Seoul, Republic of Korea). The intensity of each band was quantified using ImageJ. The intensity of HO-1 or nuclear Nrf2 was normalized to the expression of β-actin or cytosolic Nrf2, respectively, and expressed as a fold of the CTRL group.

### 2.8. Hoechst33258 Staining

Hoechst33258 was used to visualize changes in the nucleus and the formation of apoptotic bodies, which are characteristic of apoptosis, following a previous protocol [31]. Cells were seeded on a coverslip in a 12-well plate at a density of 1 × 10^5^ cells/well and incubated for 24 h. After different treatments (see Section 2.2 for details), cells were fixed with 4% paraformaldehyde (PFA) for 1 h and stained with 100 ng/mL of Hoechst33258 (Sigma-Aldrich) diluted in PBS for 5 min. After three washes with PBS, coverslips were mounted on glass slides and examined using a laser scanning confocal microscope (LSM-700, 400×). Nuclei exhibiting chromatin condensation, marginalization, or nuclear beading were considered apoptotic. More than 100 cells were counted per group, and the percentage of apoptotic nuclei was calculated relative to the total number of nuclei.

### 2.9. Flow Cytometry

Apoptosis was assessed using flow cytometry and an FITC-conjugated Annexin V apoptosis detection kit (Merck-Millipore, Kenilworth, NJ, USA), in conjunction with an automated fluorescence-activated cell sorting (FACS) device (Muse^TM^ Cell Analyzer, Merck-Millipore), following the manufacturer’s instructions. The apoptosis detection kit employed FITC-conjugated annexin-V as a marker for apoptotic cells and propidium iodide (PI) as a marker for dead cells. The FACS results provided information on the percentage of live, early apoptotic cells, late apoptotic/dead cells, and dead cells. The percentages of cells in the latter three conditions among the total cells were calculated. Data were obtained from a minimum of three independent experiments to ensure reliability.

### 2.10. Animals and Treatments

Fifty-two male C57/BL6 mice (25–30 g; 8 weeks old) were obtained from Samtako (Osan, Republic of Korea) and acclimated for 7 days with free access to water and food, maintained at a constant humidity level (40–60%) and temperature (22–24 °C), and subjected to a 12 h light/dark cycle. The mice were randomly assigned to four groups (*n* = 13 per group): ‘SO’, sham-operated; ‘VEH’, treated with vehicle (distilled water) and subjected to MCAO/R; ‘wPB-L’, treated with low dose (10 mg/kg) of wPB and subjected to MCAO/R, and ‘wPB-H’, treated with a high dose (100 mg/kg) of wPB and subjected to MCAO/R. Vehicle or wPB was administered daily via the intraoral (i.o) route for 7 days prior to MCAO/R. The animal protocol was reviewed and approved by the Institutional Animal Care and Use Committee (IACUC) at Konyang University (Daejeon, Republic of Korea), following the ethical procedures and scientific care guidelines of the National Institutes of Health Guidelines for the Care and Use of Laboratory Animals (8th Edition) [34].

### 2.11. MCAO/R

The procedure of MCAO/R was conducted under isoflurane anesthesia (2% induction, 1.5% maintenance), following a previous established protocol [35]. A midline neck incision was made, and the left common carotid artery (CCA) and the external carotid artery were tied off. A clip was placed on the internal carotid artery (ICA) to prevent bleeding. A small hole was created in the CCA, and a 7–0 nylon monofilament with a round silicon-coated tip (0.2 mm in diameter) was inserted into the hole. The silicon-coated filament was then advanced into the ICA to occlude the middle cerebral artery (MCA) until the cerebral blood flow (CBF) abruptly dropped to less than 20% of the baseline, at which point the filament insertion was stopped, and a 90 min occlusion period was initiated. After 90 min, the filament was withdrawn to initiate reperfusion. Throughout the procedure, CBF and rectal temperature (maintained at 37 ± 0.5 °C) were carefully monitored using a Doppler flowmeter (Periflux 5000; Perimed AB, Stockholm, Sweden) and a heating pad, respectively. The SO group underwent the same surgical procedure, with the exception that the filament was not sufficiently advanced to occlude the MCA.

### 2.12. Neurological Deficit Scoring

In accordance with our previous research [35], we employed neurological deficit scoring (NDS) as the initial assessment for sensorimotor deficits. Briefly, at 24 h after MCAO/R, we evaluated the sensorimotor functions of mice (*n* = 10 per group) using a scoring scale with four categories: 0 for no neurological deficit, 1 for failure to spread out the affected forepaw, 2 for unidirectional circling, 3 for falling to one side, and 4 for no voluntary movement. Two independent investigators, who were blinded to the study, conducted the NDS and calculated the average scores for each group.

### 2.13. Grip Strength Test

We used the grip strength test as the second assessment to measure sensorimotor deficits associated with fCI, following a previous study’s protocol [35]. Briefly, after completing the NDS, mice (*n* = 10 per group) were placed on a wire grid connected to a grip strength apparatus (BioSeb, Chaville, France), and their grip strength was measured as they grasped the grid with both forepaws and were gently pulled until they released their grip. The maximum force exerted was recorded in grams (g). The average values for each group were expressed as a percentage of the SO group. All tests were conducted in triplicate.

### 2.14. Rotarod Test

As a third test to assess fCI-associated sensorimotor deficits, we conducted the rotarod test in accordance with our previous study [35]. In this test, mice (*n* = 10 per group) were placed on a rotating rod with a diameter of 3 cm, using a rotarod apparatus (Ugo Basile, Varese, Italy). The time (sec) it took for them to fall from the rod was recorded. The rotation speed of the rod increased from 4 to 40 rpm over a 10 min period. Each trial ended when a mouse fell off the rod. The recorded values (sec) were averaged for each group, and all tests were conducted in triplicate.

### 2.15. Infarct Volume Measurement

To determine the extent of cerebral infarction induced by MCAO/R, 2,3,5-Triphenyltetrazolium chloride (TTC) staining was conducted following a previously established protocol [35]. Briefly, after completing all sensorimotor tests, the whole brain of each mouse (*n* = 7 per group) was harvested and sectioned into eight coronal slices with a thickness of 1 mm using a brain matrix (Zivic Instruments, Pittsburgh, PA, USA). The slices were then stained with a solution of 2% TTC (Sigma-Aldrich) diluted in PBS for 17 min at 37 °C. Subsequently, TTC-stained slices were captured using photographic imaging, and the infarcted area of each slice was quantified by a blinded observer utilizing ImageJ software. The percentage of infarcted area in each section was calculated and averaged for each experimental group.

### 2.16. Cresyl-Violet Staining and Terminal Deoxynucleotidyl Transferase dUTP Nick End Labeling Assay

To assess the extent of neuronal death and apoptosis caused by MCAO/R, we employed cresyl-violet (C-V) staining and a terminal deoxynucleotidyl transferase dUTP nick end labeling (TUNEL) assay, following the methodology from our previous publication [35]. Once all the sensorimotor tests were completed, the mice (*n* = 3 per group) were transcardially perfused with 4% PFA. Their brains were extracted, post-fixed with 4% PFA, dehydrated with graded ethanol, embedded in paraffin, and then sectioned into 5 µm slices using a microtome (RM2255, Leica, Nussloch, Germany). For C-V staining, two slides were randomly chosen from the ischemic-penumbra-bearing tissue of each mouse, deparaffinized in xylene, rehydrated with a decreasing ethanol gradient, rinsed twice in distilled water, and stained with a 0.1% C-V solution (Sigma-Aldrich). For the TUNEL assay, a commercial kit (DeadEnd^TM^ fluorometric TUNEL system, Promega, Madison, WI, USA) was used, following the manufacturer’s instructions. Two slides containing ischemic-penumbra-bearing tissue were randomly selected from each set, and the TUNEL-positive cells were counted. To analyze the results of the C-V and TUNEL staining, the stained cells were counted in at least three randomly chosen high-power fields (HPF, 400×) under a DM4 light microscope and LSM-700 laser scanning confocal microscope, respectively, and the counts were averaged for each group.

### 2.17. In Vivo ROS Measurement

We utilized dihydroethidium (DHE), an ROS probe that can be delivered in vivo, to measure ROS accumulation in the ischemic penumbra. According to the protocol described in our previous study [35], a solution of 10 mg/kg DHE (Invitrogen) in 50 µL normal saline was injected via the jugular vein into mice (*n* = 3 per group) just before the reperfusion procedure. After 2 h following MCAO/R, the brain hemisphere was collected, immersed in 30% sucrose solution, embedded in an OCT compound, and frozen using dry ice. Ischemic-penumbra-bearing sections (50 µm thick) were obtained from each mouse using a cryostat microtome (HM430, Leica) set at −21 °C. Two sections were randomly selected from each mouse and counterstained with Hoechst33258 for 5 min at 24 °C, and then mounted with a mounting medium (FluorSave^TM^, Merck-Millipore). The resulting DHE fluorescence was visualized using an LSM-700 laser scanning confocal microscope. The number of DHE-positive cells (red fluorescence) was averaged per group and expressed as a percentage of total cells (blue fluorescence), following image analysis.

### 2.18. Statistical Analyses

Data are expressed as mean ± standard deviation (SD). GraphPad Prism version 5 (GraphPad Software, La Jolla, CA, USA) was used for data analysis. The Mann–Whitney U test for non-parametric data was used to assess differences between the two groups. One-way ANOVA was performed for pairwise comparisons, followed by Tukey’s post hoc test when relevant. Statistical significance was set at *p* < 0.05.

## 3. Results

### 3.1. wPB Protects HT-22 Cells against Glutamate-Induced Excitotoxicity

**Figure 1:**
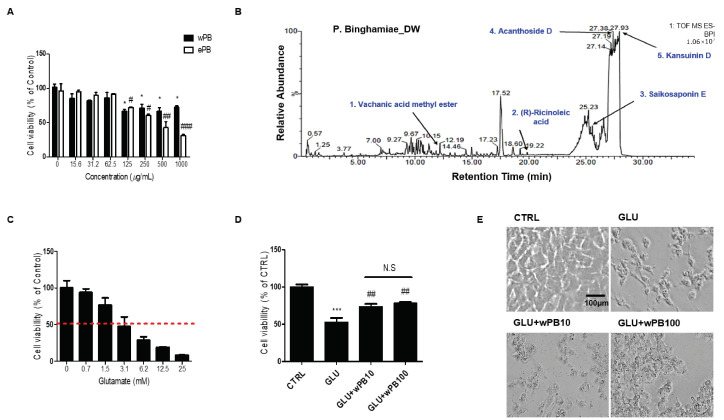
wPB protects HT-22 cells against glutamate-induced excitotoxicity. (**A**) Concentration-dependent cell viability following a 24 h of incubation with varying concentrations (0–1000 µg/mL) of water (wPB) or ethanolic (ePB) extract of *P. binghamiae* (* *p* < 0.05, ^#^ *p* < 0.05, ^##^ *p* < 0.01, and ^###^ *p* < 0.001 vs. corresponding control). (**B**) Chemical spectra of wPB obtained using liquid chromatography–mass spectrometry. (**C**) Concentration-dependent cell viability of HT-22 cells following 24 h of challenge with varying concentrations (0–25 mM) of glutamate. The interrupted red line indicates the approximate LD_50_ value. (**D**) The cell viability and (**E**) representative images of different cell groups. Values are presented as the mean ± standard deviation (*** *p* < 0.001 vs. the CTRL cells; ^##^ *p* < 0.01 vs. the GLU cells; N.S, not significant between the indicated cells). LD_50_, median lethal dose.

HT-22 cells were exposed to two different extracts, namely wPB or ePB, for 24 h, and their impact on cell viability was assessed using the MTT assay. The findings revealed that wPB had higher biocompatibility compared to ePB, with the maximum safe concentration (MSC) of wPB estimated to be between 62.5 and 125 μg/mL (Figure 1A). To identify the predominant compounds, LC-MS was employed for chemical profiling. Comparison of their retention time with an Agilent Database Library suggested that vachanic acid methyl ester, (R)-ricinoleic acid, saikosaponin E, acanthoside D, and kansuinin D were the major constituents of wPB (detector counts >150,000), arranged in ascending order of their retention times (Figure 1B and Table 1). Another cytotoxicity assay demonstrated that a 24 h incubation with 3 mM of glutamate resulted in an approximate median lethal dose (LD_50_) in our in vitro model (Figure 1C). Using the MSC value of wPB and the LD_50_ value of glutamate, the protective effect of wPB against excitotoxicity was evaluated. Consistent with expectations, the GLU cells exhibited a significant decrease in cell viability by nearly 50% (Figure 1D), accompanied by structural deterioration (Figure 1E), compared to the CTRL cells. However, the GLU + wPB10 and GLU + wPB100 cells demonstrated a significant reversal in cell viability and morphology, indicating a protective effect of wPB against excitotoxicity.

### 3.2. Antioxidant Activities of wPB Contribute to the Protection of HT-22 Cells against Glutamate-Induced Excitotoxicity

**Figure 2:**
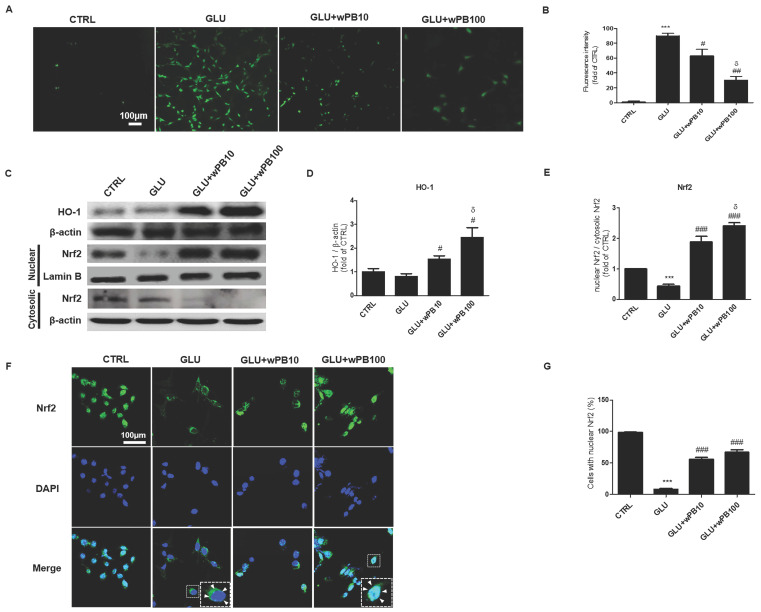
The antioxidant activity of wPB is involved in the neuroprotection against glutamate-induced excitotoxicity. (**A**) Representative images and (**B**) quantitative graph showing DCF fluorescence in HT-22 cells with different treatments. (**C**) Representative immunoblots and the corresponding densitometric bar graphs showing (**D**) HO-1 expressions as well as (**E**) nuclear Nrf2. Each band’s densities were normalized by using β-actin and cytosolic Nrf2, respectively. (**F**) Representative images and (**G**) quantitative bar graph showing the subcellular translocation of Nrf2 in cells with different treatments. In (**F**), the insets are representative portions of the same image that have been enlarged. The white arrowhead inidicated Nrf2 fluorescence. In all graphs, values are presented as the mean ± standard deviation (*** *p* < 0.001 vs. the CTRL cells; ^#^ *p* < 0.05, ^##^ *p* < 0.01 and ^###^ *p* < 0.001 vs. the GLU cells; ^δ^ *p* < 0.05 vs. the GLU + wPB10 cells). DCF, 2′,7′–dichlorodihydrofluorescein.

To determine whether antioxidant activity might be involved in the neuroprotective effect of wPB, DCF-DA was used to quantify intracellular ROS. In the GLU cells, ROS production was increased intensely by nearly 90-fold compared to the CTRL cells (Figure 2A,B). However, the increment in ROS production was significantly attenuated in the GLU + wPB10 and GLU + wPB100 cells in a concentration-dependent manner (^δ^ *p* < 0.05). To elucidate the possible contribution of HO-1 in wPB-mediated antioxidant activities, the expression level was semi-quantitatively assessed by using Western blotting (Figure 2C). In the homogenates of the GLU cells, no change was observed in the expression level of HO-1 (Figure 2D). However, the GLU + wPB10 and GLU + wPB100 cells showed significant increases in the enzyme level in a concentration-dependent manner (^δ^ *p* < 0.05). Considering the role of Nrf2 as an upstream transcriptional regulator of HO-1, the expression dynamics of Nrf2 were studied semi-quantitatively and morphologically. Western blotting revealed that the GLU cells showed a significant reduction in the relative amount of nuclear Nrf2 compared to cytosolic Nrf2, which was markedly increased in the GLU + wPB10 and GLU + wPB100 cells in a concentration-dependent manner (^δ^ *p* < 0.05, Figure 2E). Furthermore, confocal microscopy results revealed that Nrf2 in the CTRL cells was mainly located in the nucleus (Figure 2F,G). However, the majority of Nrf2 fluorescence was visible in the cytoplasm of the GLU cells. Conversely, this phenomenon was significantly reversed in both the GLU + wPB10 and GLU + wPB100 cells. These data suggest that wPB has antioxidant properties due to the increase in the nuclear translocation of Nrf2 and the subsequent upregulation of the downstream antioxidant enzyme HO-1.

### 3.3. wPB Inhibits Excitotoxicity-Associated Apoptosis of HT-22 Cells

**Figure 3:**
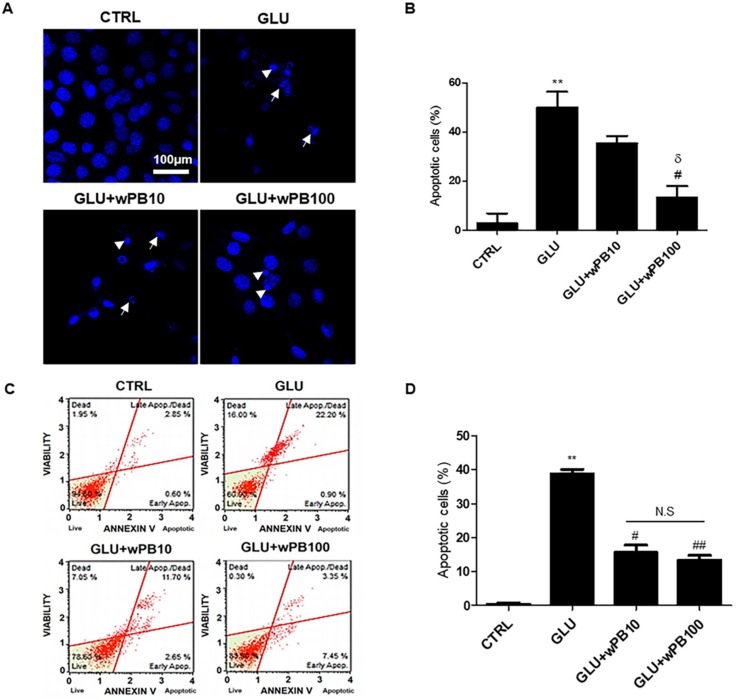
wPB inhibits the excitotoxicity-associated apoptosis of HT-22 cells. (**A**) Representative Hoechst33258-stained images and (**B**) the quantitative bar graphs of cells with different treatments. In (**A**), shrunken nuclei and apoptotic bodies are indicated by arrowhead and arrows, respectively. (**C**) Representative dot plot diagrams showing apoptosis-ongoing cells and (**D**) quantitative bar graphs obtained by using AnnexinV-FITC/PI-based FACS. In all graphs, values are presented as the mean ± standard deviation (** *p* < 0.01 vs. the CTRL cells; ^#^ *p* < 0.05 and ^##^ *p* < 0.01 vs. the GLU cells; ^δ^ *p* < 0.05 vs. the GLU + wPB10 cells; N.S, not significant between the indicated cells). FITC, fluorescein isothiocyanate; PI, propidium iodide; FACS, fluorescence-activated cell sorting.

Due to the role of ROS-induced oxidative damage in triggering neuronal apoptosis in fCI pathology, we investigated the potential anti-apoptotic effect of wPB. Results from Hoechst33258 staining demonstrated a significantly higher percentage of apoptotic cells, characterized by shrunken nuclei (indicated by the arrowhead in Figure 3A) and apoptotic bodies (indicated by arrow), in the GLU cells compared to the CTRL cells (Figure 3B). However, the percentage of apoptotic cells was notably reduced in GLU + wPB100 cells. This finding was further supported by Annexin V-FITC/PI-based FACS results, which revealed an increase in the percentage of apoptotic and dead cells in the GLU cells compared to that in the CTRL cells (Figure 3C,D). In contrast, the GLU + wPB10 and GLU + wPB100 cells showed a significant reduction in this value. These results indicate that wPB treatment may protect neurons from excitotoxicity-induced apoptosis.

### 3.4. wPB Can Minimize MCAO/R-Triggered Infarct Volume and Neuronal Death

**Figure 4:**
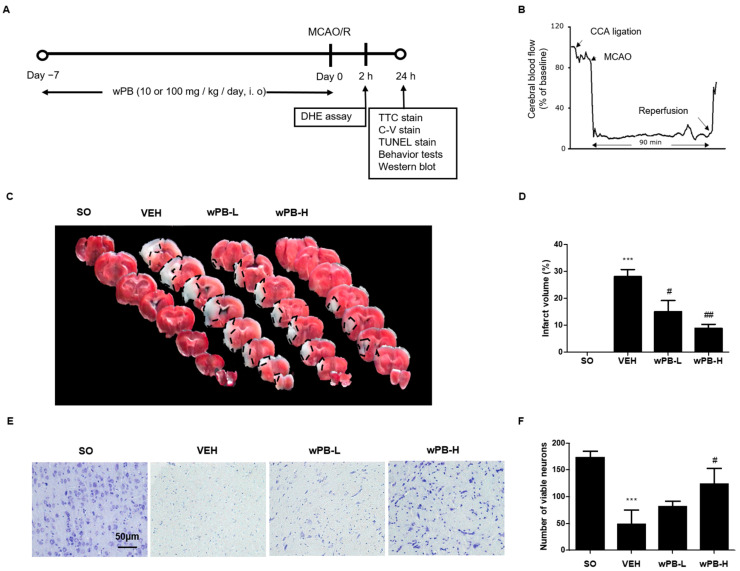
wPB can minimize infarct volume and neuronal death in mice with focal cerebral ischemia. (**A**) Timelines of the in vivo experimental protocols and (**B**) representative Doppler flowmetry showing time-dependent changes in cerebral blood flow values in mice during the MCAO/R operation. (**C**) Representative photographs showing the TTC-stained brain serial sections and (**D**) quantitative graphs showing the % area of infarction of different groups. Upon TTC staining, infarcted areas generally appear white. (**E**) A representative image of C-V-stained ischemic penumbra tissue and (**F**) quantitative graph showing the average number of viable neurons of the different groups. In all graphs, values are presented as the mean ± standard deviation (*** *p* < 0.001 vs. the SO group; ^#^ *p* < 0.05; and ^##^ *p* < 0.01 vs. the VEH group). MCAO/R, middle cerebral artery occlusion and reperfusion; TTC, 2,3,5-Triphenyltetrazolium chloride; C-V, cresyl-violet.

To extend the findings from in vitro to in vivo experiments, we employed MCAO/R surgery to replicate clinical fCI. Mice were administered wPB at either a low dose (wPB-L group, 10 mg/kg) or a high dose (wPB-H group, 100 mg/kg) via an i.o. route for 7 days before the surgery, as depicted in Figure 4A. Within 24 h after the MCAO/R surgery, various morphological and behavioral analyses were performed on the mice groups, including those treated with vehicle for 7 days followed by MCAO/R (VEH group) or sham operation (SO group). To ensure the reliability of the MCAO/R procedure, we monitored CBF values using Doppler flowmetry (Figure 4B). TTC-stained tissues revealed no visible areas of infarction in the SO group, as expected (Figure 4C,D). In contrast, the VEH group exhibited enlarged infarcted areas at 24 h after MCAO/R, which were significantly reduced in both the wPB-L and wPB-H groups. This observation was consistent with a decrease in viable neurons in the ischemic penumbra tissue of the VEH group, as evident from C-V staining (Figure 4E,F). However, the wPB-H group demonstrated a significant preservation in the number of viable neurons in the ischemic penumbra. These findings suggest that preoperative administration of wPB can attenuate fCI-induced infarct volume and neuronal death in the ischemic penumbra.

### 3.5. wPB Can Attenuate fCI-Associated Sensorimotor Deficits

**Figure 5:**
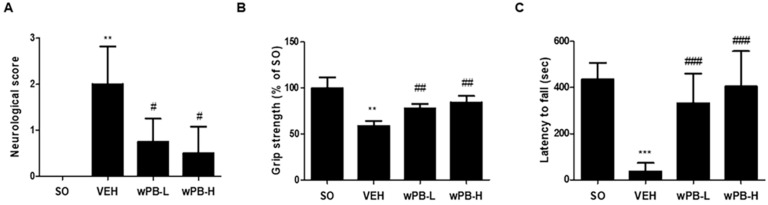
wPB can attenuate sensorimotor deficits induced by focal cerebral ischemia. (**A**) Quantitative graph showing the results of neurological deficit scoring, (**B**) grip strength test, and (**C**) rotarod test in different groups performed at 24 h after MCAO/R. In all graphs, values are presented as the mean ± standard deviation (** *p* < 0.01 and *** *p* < 0.001 vs. the SO group; ^#^ *p* < 0.05, ^##^ *p* < 0.01, and ^###^ *p* < 0.001 vs. the VEH group). MCAO/R, middle cerebral artery occlusion and reperfusion.

Three different behavioral tests were conducted at 24 h after MCAO/R to assess the modulatory potential of wPB in fCI-associated sensorimotor deficits. First, NDS demonstrated prominent deficits in the VEH group (Figure 5A). However, both the wPB-L and wPB-H groups exhibited significantly improved neurological function compared to the VEH group. Second, the grip strength test results showed that the VEH group had significantly lower strength than the SO group (Figure 5B). Conversely, both the wPB-L and wPB-H groups exhibited markedly increased strength. Third, the rotarod test revealed significantly reduced latencies to fall from the rotating drum in the VEH group compared to the SO group (Figure 5C). In contrast, both the wPB-L and wPB-H groups remained on the rotating drum for a much longer duration than the VEH group. Although we failed to observe dose-dependence in the three tests, these findings demonstrate that wPB can attenuate the sensorimotor deficits typically observed in fCI mice.

### 3.6. wPB Protects Neurons against fCI via Antioxidative and Antiapoptotic Properties

**Figure 6:**
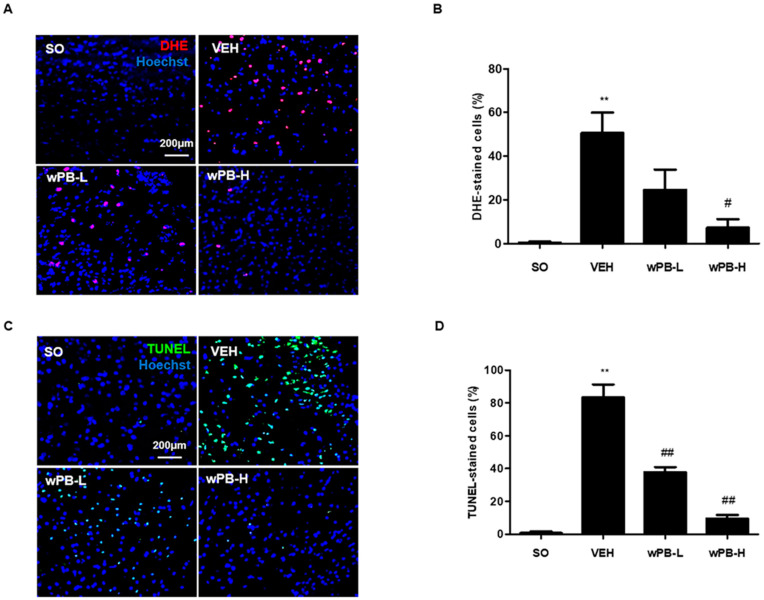
wPB protects mice against focal cerebral ischemia via antioxidative and antiapoptotic properties. (**A**) A representative image showing DHE-fluorescence (red) in the ischemic penumbra of different groups acquired at 2 h after MCAO/R and (**B**) quantitative graphs showing the number of DHE-positive cells. (**C**) A representative image showing TUNEL-stained cells (green) in the ischemic penumbra of different groups acquired at 24 h after MCAO/R and (**D**) the quantitative graphs. In (**A**,**C**), Hoechst33258 was used to stain cell nuclei. In both graphs, values are presented as the mean ± standard deviation (** *p* < 0.01 vs. the SO group; ^#^ *p* < 0.05 and ^##^ *p* < 0.01 vs. the VEH group). DHE, dihydroethidium; TUNEL, terminal deoxynucleotidyl transferase dUTP nick end labeling; MCAO/R, middle cerebral artery occlusion and reperfusion.

Having established that wPB can reduce fCI-associated phenotypes, we conducted further studies to investigate whether wPB’s antioxidant and anti-apoptotic effects, previously demonstrated in vitro, might also contribute to neuroprotection in vivo. For this purpose, we administered DHE, an in vivo deliverable ROS marker, to mice via an intravenous route at the end of MCAO/R, and their brains were obtained at 2 h after surgery. As shown in Figure 6A,B, ROS accumulation (red fluorescence) was significantly higher in the ischemic penumbra tissue of the VEH group compared to the SO group. However, both the wPB-L and wPB-H groups exhibited a substantial reduction in DHE fluorescence when compared to the VEH group. Subsequently, using tissue slides from the ischemic penumbra, we quantified neuronal apoptosis by conducting a TUNEL assay. While TUNEL-positive apoptotic cells were scarcely detectable in the SO group, as expected, the VEH group showed a significant increase in the number of TUNEL-positive cells (Figure 6C,D). In contrast, both the wPB-L and wPB-H groups exhibited a noteworthy reduction in TUNEL fluorescence when compared to the VEH group. These results suggest that both the antioxidant and anti-apoptotic effects of wPB contribute to in vivo protection against fCI.

## 4. Discussion

The development of fCI begins with a permanent or temporary compromise in blood flow to a specific region of the brain, leading to a deprivation of nutrients and oxygen. This results in energy failure and the accumulation of toxic byproducts [7,11]. Among these toxic byproducts, ROS, such as hydroxyl radicals, superoxide radicals, and peroxyl radicals, have been identified as crucial molecules that can cause neuronal death [32]. Normally, there is a balance between ROS production and elimination in healthy conditions, which is maintained by intracellular antioxidant enzymes, primarily including HO-1 [35,36,37]. However, during fCI, multiple pathological pathways trigger excessive ROS production in neurons, leading to structural damage to various intracellular organelles, such as lipid-rich cell membranes, mitochondria, and DNA, posing a threat to neuronal survival [38]. Based on this concept, enhancing intracellular HO-1 levels to counteract the overproduction of ROS could be a potential therapeutic strategy against fCI.

This study revealed that wPB exerts neuroprotective effects against fCI and these effects are partly attributed to the upregulation of the Nrf2/HO-1 pathway. Due to its role as a master regulator of various antioxidative enzymes, including HO-1 [39], Nrf2 has been recognized as a promising therapeutic target for central nervous system (CNS) diseases [40,41]. In healthy cells, cytosolic Nrf2 is bound to Kelch-like ECH-associated protein 1 (Keap1), which prevents its translocation into the nucleus. However, under specific stimuli that induce conformational changes in the Nrf2-Keap1 complex, Nrf2 is able to enter the nucleus and bind to antioxidant-related elements (AREs) in the promoter regions of downstream genes [42,43]. While previous research has accumulated evidence on the modulatory effects of different marine algal species and their constituents on Nrf2/HO-1 dynamics [36,39], this study is the first, to the best of our knowledge, to demonstrate that supplementation with wPB can protect against fCI-induced neuronal injury through activation of this pathway.

Marine algal species, traditionally used as dietary ingredients in Eastern Asian countries like Japan, China, and Republic of Korea, have been reported to contain health-promoting components such as dietary fibers, polysaccharides, proteins, vitamins, minerals, polyphenols, and sterols [44]. Remarkably, algae are expected to produce compounds that possess either direct antioxidant properties or the ability to upregulate endogenous antioxidant enzymes as an evolutionary adaptation to hostile environments characterized by extreme temperatures, acidities, pressures, and limited substrates [20]. Among algae, PB is a brown alga found in warm to temperate coastal areas of the Pacific Ocean. It belongs to the family Scytosiphonaceae and is typically found in the upper to mid-tidal zones. Although the species’ life cycle, morphologic variability, photosynthetic properties, and chemical composition have been well-described [43], information about its bioactive functions is limited.

In this study, it was discovered that wPB had protective effects in an experimental model of fCI, both in vitro and in vivo. Specifically, coincubation with wPB was found to rescue HT-22 cells, a neuronal cell line, from various pathological changes associated with excitotoxicity in vitro. Furthermore, wPB supplementation was shown to alleviate fCI-associated symptoms in vivo. In addition to investigating the neuroprotective effect and underlying mechanism of wPB, this study also aimed to identify the compounds present in wPB. Although further analyses using chemistry-based methods are needed, LC-MS results revealed that vachanic acid methyl ester, (R)-ricinoleic acid, saikosaponin E, acanthoside D, and kansuinin D were among the major constituents, listed in increasing order of retention time. While information on the biological effects of these individual compounds is largely limited, (R)-ricinoleic acid and acanthoside D have been relatively well-studied. For example, (R)-ricinoleic acid, a prominent component of castor oil, has been shown to exert inhibitory effects on acute or subchronic inflammation models induced by carrageenan or histamine in mice and guinea pigs via modulation of the levels of cyclooxygenase-2 and nitric oxide, respectively [45]. Acanthoside D, originally isolated from the acanthopanax genus, has been reported to possess various functions, including anti-tumor, anti-inflammatory, and anti-myocardial infarction effects [46,47,48], possibly mediated through the downregulation of inflammatory factor release [49]. The identification of these two compounds in wPB through LC-MS analysis may suggest the potential of PB as a novel biofunctional food for the prevention or treatment of inflammation-based human diseases.

Nevertheless, we cannot rule out the possibility that the biologic effects of other constituents were involved in the neuroprotection conferred by PB. For example, heterogeneous groups of polysaccharides, including fucoidan and laminarin, account for more than 50% of the total dry weight of brown algae and can reach up to 70% in some species. In fact, fucoidan has been reported to possess protective effects against experimental models of hepatic ischemia–reperfusion injury and global cerebral ischemia [50,51]. Furthermore, laminarin was reported to protect gerbils from forebrain ischemia by reducing oxidative stress and neuroinflammation [52]. In order to facilitate the development of novel pharmaceuticals through the quantitative analysis of the neuroprotective efficacy of individual components within PB, a sophisticated integration of diverse and contemporary chemical analysis methods with precise molecular biology experiments is imperative.

Despite uncovering several novel findings, this study has some limitations. Firstly, wPB was administered preoperatively, which precludes determining its potential therapeutic effects after fCI occurs. To address this, future studies with binary protocols involving both preoperative and postoperative treatments are warranted. Secondly, although this study somewhat elucidated the therapeutic mechanisms underlying wPB-induced fCI protection, focusing on the modulation of oxidative burden, it is possible that wPB may also affect other cell components in the ischemic brain beyond neurons. Recent research has emphasized the importance of the neurogliovascular unit (NGVU) in fCI pathogenesis and drug discovery. This unit encompasses a network of neurons, glia, and endothelial cells that functionally interact within the brain microenvironment [53,54]. As fCI is known to involve dysfunctions in the three primary cell types of the NGVU, including neuronal apoptosis, neuroinflammation, and blood–brain barrier breakdown, a more comprehensive approach involving multicellular systems of NGVU is needed in future studies to gain deeper insight into the neuroprotective potential of wPB in fCI.

## 5. Conclusions

This study revealed that wPB has protective effects in an experimental model of fCI both in vitro and in vivo. Coincubation with wPB rescued a neuronal cell line from various pathological changes associated with excitotoxicity in vitro. wPB supplementation alleviated fCI-related symptoms in fCI mice in vivo. The neuroprotective effects of wPB were attributed to the upregulation of the Nrf2/HO-1 pathway, suggesting that wPB exerts its therapeutic effects against fCI through its antioxidative properties.

## Figures and Tables

**Table 1:** Mass spectrometric data (retention time, molecular formula, molecular weight, and charge-to-mass ratio) of the verified compounds.

	Retention Time (min)	Molecular Formula	Molecular Weight (Da)	[M-H]^−^ (*m*/*z*)	Compound
1	12.20	C_16_H_26_O_3_	266.2	311.1865	Vachanic acid methyl ester
2	19.78	C_18_H_34_O_3_	298.5	301.3041	(R)-ricinoleic acid
3	25.55	C_34_H_46_O_18_	764.5	763.4675	Saikosaponin E
4	27.38	C_34_H_46_O_18_	742.7	789.4834	Acanthoside D
5	27.90	C_41_H_47_NO_15_	793.3	792.2906	Kansuinin D

## Data Availability

The datasets generated and analyzed during the current study are available from the corresponding authors on request.

## References

[1] Beal C.C. (2010). Gender and stroke symptoms: A review of the current literature. J. Neurosci. Nurs..

[2] Heron M. (2007). Deaths: Leading causes for 2004. Natl. Vital. Stat. Rep..

[3] Besancon E., Guo S., Lok J., Tymianski M., Lo E.H. (2008). Beyond NMDA and AMPA glutamate receptors: Emerging mechanisms for ionic imbalance and cell death in stroke. Trends Pharmacol. Sci..

[4] Ramon R., Julio César G. (2016). Chapter 2—Excitotoxicity and Oxidative Stress in Acute Stroke. Ischemic Stroke.

[5] Ouyang Y.B., Voloboueva L.A., Xu L.J., Giffard R.G. (2007). Selective dysfunction of hippocampal CA1 astrocytes contributes to delayed neuronal damage after transient forebrain ischemia. J. Neurosci..

[6] Xu L., Emery J.F., Ouyang Y.B., Voloboueva L.A., Giffard R.G. (2010). Astrocyte targeted overexpression of Hsp72 or SOD2 reduces neuronal vulnerability to forebrain ischemia. Glia.

[7] Khatri R., McKinney A.M., Swenson B., Janardhan V. (2012). Blood-brain barrier, reperfusion injury, and hemorrhagic transformation in acute ischemic stroke. Neurology.

[8] Castilho R.F., Hansson O., Ward M.W., Budd S.L., Nicholls D.G. (1998). Mitochondrial control of acute glutamate excitotoxicity in cultured cerebellar granule cells. J. Neurosci..

[9] Orellana-Urzua S., Rojas I., Libano L., Rodrigo R. (2020). Pathophysiology of Ischemic Stroke: Role of Oxidative Stress. Curr. Pharm. Des..

[10] Khoshnam S.E., Winlow W., Farzaneh M., Farbood Y., Moghddam H.F. (2017). Pathogenic mechanisms following ischemic stroke. Neurol. Sci..

[11] Song K., Li Y., Zhang H., An N., Wei Y., Wang L., Tian C., Yuan M., Sun Y., Xing Y. (2020). Oxidative Stress-Mediated Blood-Brain Barrier (BBB) Disruption in Neurological Diseases. Oxidative Med. Cell. Longev..

[12] Ridder D.A., Schwaninger M. (2009). NF-kappaB signaling in cerebral ischemia. Neuroscience.

[13] Culmsee C., Zhu C., Landshamer S., Becattini B., Wagner E., Pellecchia M., Blomgren K., Plesnila N. (2005). Apoptosis-inducing factor triggered by poly(ADP-ribose) polymerase and Bid mediates neuronal cell death after oxygen-glucose deprivation and focal cerebral ischemia. J. Neurosci..

[14] Huang X., He D., Pan Z., Deng J. (2021). Reactive-oxygen-species-scavenging nanomaterials for resolving inflammation. Mater. Today Bio..

[15] Polaka S., Katare P., Pawar B., Vasdev N., Gupta T., Rajpoot K., Sengupta P., Tekade R.K. (2022). Emerging ROS-Modulating Technologies for Augmentation of the Wound Healing Process. ACS Omega.

[16] Casas A.I., Nogales C., Mucke H.A.M., Petraina A., Cuadrado A., Rojo A.I., Ghezzi P., Jaquet V., Augsburger F., Dufrasne F. (2020). On the Clinical Pharmacology of Reactive Oxygen Species. Pharmacol. Rev..

[17] Poljsak B., Šuput D., Milisav I. (2013). Achieving the balance between ROS and antioxidants: When to use the synthetic antioxidants. Oxid. Med. Cell Longev..

[18] Cochemé H., Murphy M. (2010). Can antioxidants be effective therapeutics? Curr Opin Investig Drugs. Curr. Opin. Investig. Drugs.

[19] Ghosh S., Sarkar T., Pati S., Kari Z.A., Edinur H.A., Chakraborty R. (2022). Novel Bioactive Compounds from Marine Sources as a Tool for Functional Food Development. Front. Mar. Sci..

[20] Macedo M.W.F.S., Cunha N.B.d., Carneiro J.A., Costa R.A., Alencar S.A., Cardoso M.H., Franco O.L., Dias S.C. (2021). Marine Organisms as a Rich Source of Biologically Active Peptides. Front. Mar. Sci..

[21] Catanesi M., Caioni G., Castelli V., Benedetti E., Angelo M., Cimini A. (2021). Benefits under the Sea: The Role of Marine Compounds in Neurodegenerative Disorders. Mar. Drugs.

[22] Morris J.J., Kirkegaard R., Szul M.J., Johnson Z.I., Zinser E.R. (2008). Facilitation of robust growth of Prochlorococcus colonies and dilute liquid cultures by "helper" heterotrophic bacteria. Appl. Environ. Microbiol..

[23] Lee H.S., Kwon K.K., Kang S.G., Cha S.S., Kim S.J., Lee J.H. (2010). Approaches for novel enzyme discovery from marine environments. Curr. Opin. Biotechnol..

[24] Nalini S., Sandy Richard D., Mohammed Riyaz S.U., Kavitha G., Inbakandan D. (2018). Antibacterial macro molecules from marine organisms. Int. J. Biol. Macromol..

[25] Khalifa S.A.M., Elias N., Farag M.A., Chen L., Saeed A., Hegazy M.E., Moustafa M., Washed A.A., Mousawi S.M., Musharraf S. (2019). Marine Natural Products: A Source of Novel Anticancer Drugs. Mar. Drugs.

[26] Sagar S., Kaur M., Minneman K.P. (2010). Antiviral lead compounds from marine sponges. Mar. Drugs.

[27] Buchanan J. (2007). The Crustose Brown Algae of New Zealand: A Taxonomic Study. Master’s Thesis.

[28] Kang S.I., Jin Y.J., Ko H.C., Choi S.-Y., Hwang J.H., Whang I., Kim M.H., Shin H.S., Jeong H.B., Kim S.J. (2008). Petalonia improves glucose homeostasis in streptozotocin-induced diabetic mice. Biochem. Biophys. Res. Commun..

[29] Yang E.J., Moon J.Y., Kim M.J., Kim D.S., Lee W.J., Lee N.H., Hyun C.G. (2010). Anti-inflammatory Effect of Petalonia binghamiae in LPS- Induced Macrophages is Mediated by Suppression of iNOS and COX-2. Int. J. Agric. Biol..

[30] Kang J.S., Choi I.W., Han M.H., Lee D.S., Kim G.Y., Hwang H.J., Kim B.W., Kim C.M., Yoo Y.H., Choi Y.H. (2015). The Cytoprotective Effect of Petalonia binghamiae Methanol Extract against Oxidative Stress in C2C12 Myoblasts: Mediation by Upregulation of Heme Oxygenase-1 and Nuclear Factor-Erythroid 2 Related Factor 2. Mar. Drugs.

[31] Jeong J.H., Kim S.H., Park M.N., Park J.Y., Park H.Y., Song C.E., Moon J.H., Choi A.L., Kim K.D., Lee N.S. (2021). Water Extract of Mixed Mushroom Mycelia Grown on a Solid Barley Medium Is Protective against Experimental Focal Cerebral Ischemia. Curr. Issues Mol. Biol..

[32] Wei E.P., Kontos H.A., Beckman J.S. (1996). Mechanisms of cerebral vasodilation by superoxide, hydrogen peroxide, and peroxynitrite. Am. J. Physiol..

[33] Allen C.L., Bayraktutan U. (2009). Oxidative stress and its role in the pathogenesis of ischaemic stroke. Int. J. Stroke.

[34] Committee for the Update of the Guide for the Care and Use of Laboratory Animals, Institute for Laboratory Animal Research, Division on Earth and Life Studies (2011). Guide for the Care and Use of Laboratory Animals.

[35] Lee J., Jeong J., Jeong Y.-G., Kim D.-K., Lee N.S., Na C.S., Doh E.S., Han S.Y. (2019). Platycarya strobilacea leaf extract protects mice brain with focal cerebral ischemia by antioxidative property. Anat. Cell Biol..

[36] Margaill I., Plotkine M., Lerouet D. (2005). Antioxidant strategies in the treatment of stroke. Free Radic. Biol. Med..

[37] Jiang S., Deng C., Lv J., Fan C., Hu W., Di S., Yan X., Ma Z., Liang Z., Yang Y. (2017). Nrf2 Weaves an Elaborate Network of Neuroprotection Against Stroke. Mol. Neurobiol..

[38] Jia J., Jin H., Nan D., Yu W., Huang Y. (2021). New insights into targeting mitochondria in ischemic injury. Apoptosis.

[39] Parente M., Neto A.I., Fletcher R. (2003). Morphology and life history studies of *Endarachne binghamiae* (*Scytosiphonaceae*, *Phaeophyta*) from the Azores. Aquat. Bot..

[40] Zhang C., Shu L., Kong A.N. (2015). MicroRNAs: New players in cancer prevention targeting Nrf2, oxidative stress and inflammatory pathways. Curr. Pharmacol. Rep..

[41] Johnson J.A., Johnson D.A., Kraft A.D., Calkins M.J., Jakel R.J., Vargas M.R., Chen P.C. (2008). The Nrf2-ARE pathway: An indicator and modulator of oxidative stress in neurodegeneration. Ann. N. Y. Acad. Sci..

[42] Hardingham G.E., Lipton S.A. (2011). Regulation of neuronal oxidative and nitrosative stress by endogenous protective pathways and disease processes. Antioxid. Redox Signal..

[43] Kaspar J.W., Niture S.K., Jaiswal A.K. (2009). Nrf2:INrf2 (Keap1) signaling in oxidative stress. Free Radic. Biol. Med..

[44] Hiraganahalli B.D., Chinampudur V.C., Dethe S., Mundkinajeddu D., Pandre M.K., Balachandran J., Agarwal A. (2012). Hepatoprotective and antioxidant activity of standardized herbal extracts. Pharmacogn. Mag..

[45] Ambreen Bano A.H., Swati Sharma Snober S. (2023). Mir. Exploring Poisonous Plants Medicinal Values, Toxicity Responses, and Therapeutic Uses.

[46] Matos P., Batista M.T., Figueirinha A. (2022). A review of the ethnomedicinal uses, chemistry, and pharmacological properties of the genus Acanthus (Acanthaceae). J. Ethnopharmacol..

[47] Pabis S., Kula J. (2016). Synthesis and Bioactivity of (R)-Ricinoleic Acid Derivatives: A Review. Curr. Med. Chem..

[48] Vieira C., Fetzer S., Sauer S.K., Evangelista S., Averbeck B., Kress M., Reeh P.W., Cirillo R., Lippi A., Maggi C. (2001). Pro- and anti-inflammatory actions of ricinoleic acid: Similarities and differences with capsaicin. Naunyn Schmiedebergs Arch. Pharmacol..

[49] Yang L., Li D., Zhuo Y., Zhang S., Wang X., Gao H. (2016). Protective Role of Liriodendrin in Sepsis-Induced Acute Lung Injury. Inflammation.

[50] Li X.J., Ye Q.F. (2015). Fucoidan reduces inflammatory response in a rat model of hepatic ischemia-reperfusion injury. Can. J. Physiol. Pharmacol..

[51] Slim C., Nassrallah H., Zaouali M.A., Amara F., Majdoub H., Morin D., Abdennebi H.B. (2022). Fucoidan alleviates the mitochondria and endoplasmic reticulum stresses in ischemic rat livers. Phytomedicine Plus.

[52] Park J.H., Ahn J.H., Lee T.K., Par C.W., Kim B., Lee J.-C., Kim D.W., Shin M.G., Cho J.H., Lee C.-H. (2020). Laminarin Pretreatment Provides Neuroprotection against Forebrain Ischemia/Reperfusion Injury by Reducing Oxidative Stress and Neuroinflammation in Aged Gerbils. Mar. Drugs.

[53] Cai W., Zhang K., Li P., Zhu L., Xu J., Yang B., Hu X., Lu Z., Chen J. (2017). Dysfunction of the neurovascular unit in ischemic stroke and neurodegenerative diseases: An aging effect. Ageing Res. Rev..

[54] Kugler E.C., Greenwood J., MacDonald R.B. (2021). The “Neuro-Glial-Vascular” Unit: The Role of Glia in Neurovascular Unit Formation and Dysfunction. Front. Cell Dev. Biol..

